# Experiences of Dutch ambulance nurses in emergency care for patients with acute manic and/or psychotic symptoms: A qualitative study

**DOI:** 10.1111/ppc.12691

**Published:** 2020-12-03

**Authors:** Thea H. Daggenvoorde, Josette M. van Klaren, Harm J. Gijsman, Hester Vermeulen, Peter J. J. Goossens

**Affiliations:** ^1^ Center for Bipolar Disorders Dimence Mental Health Deventer The Netherlands; ^2^ Radboud Institute for Health Sciences IQ Healthcare Radboud University Medical Center Nijmegen The Netherlands; ^3^ Institute of Nursing studies University of Applied Sciences Utrecht The Netherlands; ^4^ Clinical Health Science University of Utrecht Utrecht The Netherlands; ^5^ Division of Emergency Mental Health Care Dimence Mental Health Deventer The Netherlands; ^6^ Department of Public Health and Primary Care, Faculty of Medicine and Health Sciences University Centre for Nursing and Midwifery, Ghent University Ghent Belgium

**Keywords:** ambulance nurse, emergency care, manic, mental health, psychotic

## Abstract

**Purpose:**

To explore the experiences of ambulance nurses in emergency care of patients with acute manic and/or psychotic symptoms.

**Methods:**

In this qualitative study, 14 interviews were conducted and analyzed using thematic analysis according to Braun and Clarke (2006).

**Findings:**

Psychiatric emergency care causes stress and uncomfortable feelings for ambulance nurses due to a lack of information on the patients, being alone with the patient in a small place and the unpredictability of the situation.

**Practice implications:**

More information about the specific patient, education, and good collaboration with other professionals could improve care.

## 1 INTRODUCTION

Manic and/or psychotic symptoms could lead to a mental health crisis, defined as “*any situation in which a person's behavior puts them at risk of hurting themselves or others and/or prevents them from being able to care for themselves or function effectively in the community*.”^1^

Psychosis is characterized by the presence of hallucinations without insight, delusions, or both.^2^ Mania is a period of elevated, tense moods, and increased activity or energy during a period of at least 1 week for most of the day.^2^ A large portion of patients who need psychiatric emergency care have manic and/or psychotic symptoms. Barker et al.^3^ report 22% patients with bipolar and schizoaffective disorders and schizophrenia. Mulder et al.^4^ describe in their study 37% patients with psychotic disorders and 29% patients in a manic state.

During a psychiatric crisis care takes place in a chain of care providers with different tasks and angles of approach. Mobile crisis teams aim to offer ambulatory psychiatric care for patients in a psychiatric crisis and their family.^5^, ^6^, ^7^ Police officers are involved in case of unsafe situations.^8^ In the case of compulsory hospitalization, ambulance services are responsible to transport the patient to a psychiatric hospital.

In an earlier study, experiences of patients with acute manic and/or psychotic symptoms and their family members with psychiatric emergency care were explored.^9^ Communication and cooperation was experienced as difficult in several cases. Personal crisis plans were not always used. Stigma was felt, especially when police‐assistance was needed. Family members often felt powerless to handle the crisis. A calm, understanding attitude of the professionals was appreciated.^9^

As mentioned, ambulance services are one of the care providers that deliver psychiatric emergency care. Dealing with cases of mental illness is a significant component of ambulance nurses’ working life.^10^, ^11^ In The Netherlands registered nurses are only permitted to work on an ambulance if they succesfully completed an additional 18 month ambulance nurse training. To our knowledge, few studies^10^, ^11^, ^12^ have been published about experiences of ambulance nurses with psychiatric emergency care. Ambulance nurses were frustrated by “filling the gaps for other healthcare services” in caring for patients with mental health problems (Prener & Lincoln,^12^ p. 617). Another study described the working relationship between ambulance nurses and mobile crisis teams as ineffective. These teams extended their scene time and were often difficult to contact. Ambulance nurses emphasized a need for clear policy relating to the interaction between mobile crisis teams and ambulance nurses.^11^ The results of previous studies cannot be generalized to Dutch psychiatric emergency care due to differences in legislation, healthcare processes, and culture. In this study, the experiences of Dutch ambulance nurses in emergency care for patients with acute manic and/or psychotic symptoms are explored. It is important to create a broader view on emergency care for patients with acute manic and/or psychotic symptoms to find leads to improve the quality of care.

## 2 METHODS

### 2.1 Design

An explorative, qualitative, generic design was used because little knowledge is available about the experiences of ambulance nurses in psychiatric emergency care.^13^

### 2.2 Participants and recruitment

**Figure 1:**
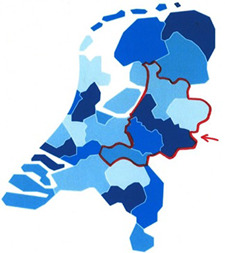
Participating regional ambulance services [Color figure can be viewed at wileyonlinelibrary.com]

Ambulance nurses from five regional ambulance services (RAVs) in the eastern Netherlands (see Figure 1. Participating Regional Ambulance Services) were selected by a convenience sampling strategy.^14^ Participants in this study had to be involved in emergency care of patients who experienced acute manic and/or psychotic symptoms at least five times in the last 3 years. The sample size of this study was determined by theoretical saturation.^15^ The study was conducted from January 2019 to July 2019.

The manager of each selected RAV received an explanation of the study by email and informed the ambulance nurses. Interested nurses were asked to email the principal researcher. They received an information leaflet and had the opportunity to ask questions. After 1 week, they were contacted to ask if they still were interested to participate in this study. All consented to participate. Time and location for the interview were arranged. Before the start of the actual interview, written informed consent was obtained.

### 2.3 Data collection

Unstructured, nonstandardized interviews were used to follow thoughts and interests of participants.^15^ Each interview started with collecting demographic characteristics followed by the general question: “*What are your most memorable experiences in the care for patients with acute manic and/or psychotic symptoms?*” An aide‐mémoire (Table 1) was used to guide the participants back to the topic of study if distracted during the interview. This aide‐mémoire was based on the literature and was presented to an experienced nurse researcher working in a mobile crisis team and by a physician assistant employed in an RAV. Afterwards, the aide‐mémoire was discussed in the research team, and after consensus was reached it was validated for this study. A pilot interview was conducted with an ambulance nurse, transcribed verbatim, and discussed with the coordinating researcher to receive feedback on the interview style and to evaluate the aide‐mémoire.

**Table 1:** Aide‐mémoire

Starting Question: *What are your most memorable experiences in the care for patients with acute manic and/or psychotic symptoms?*
Contact with the patient
Contact with family members
Contact with professionals of the mobile crisis team
Information transfer between ambulance nurses and professionals of the mobile crisis team, beforehand and during the contact
Collaboration with the police
Experiences before transport
Experiences during the transport
Experiences during transmission to the psychiatric hospital
Following emergency care

All individual interviews were conducted at the work location of the participants by one researcher (second author). The research team consisted of a clinical health science student who has work experience in mental health nursing (second author); a researcher who also has experience as nurse in a mobile crisis team (first author); an MD with experience as psychiatrist in a mobile crisis team (third author); a coordinating researcher, professor, and nurse specialist in mental health care (last author); and a professor in nursing science, nurse and, clinical epidemiologist (fourth author). To enhance confirmability, the second author critically reflected on her own assumptions by writing them down before the start of the study.

Field notes were written down by the researcher directly after the interviews in a logbook. All interviews were audio recorded and transcribed verbatim, and checked for errors. Reflections and field notes were regularly discussed with the research team to stay aware of prejudices.^15^

### 2.4 Data analysis

Thematic analysis according to Braun and Clarke^14^ was used. After the first three interviews, initial codes were generated by the second and last author individually. These initial codes were discussed to achieve consensus. The other interviews were coded by the second author, and initial and new codes were discussed within the research team. Next the research team identified and discussed subthemes. In an ongoing process checks were made whether the themes could be traced back to the original data set. Thereafter, main themes were identified by the research team. Memos were made during analysis, containing thoughts and ideas about data and the clustering of codes. This process of identifying and clustering main themes was repeated several times before consensus was reached within the team. During the continuous process of gathering and analyzing data the research team was aware of the point where no further dimensions, insights or themes were found. After the twelfth interview two additional interviews were held to be sure that data saturation was achieved.^15^ The use of researcher triangulation and peer debriefing during the analysis enhanced the creditability and conformability. Member checking was used to enhance the credibility.^15^, ^16^ All fourteen participants received a first concept of the findings, eight of them reacted: they agreed with the description. Two of them had an addition about difficulties in getting enough information of the mobile crisis team and their wish to evaluate the given care in a specific situation. This information was added to the findings. NVivo, version 12, QRS international, Australia, was used to organize the data.

### 2.5 Ethical considerations

This study was conducted according to the principles of the Declaration of Helsinki.^17^ Formal approval from the Dutch Central Committee on Research Involving Human Subjects was not needed because participants were not subject to procedures or required to follow rules of behavior (http://www.ccmo.nl/en).

The research proposal was assessed by the scientific committee of the mental health organization where the coordinating researcher works. All data were anonymized and stored on a secure data storage for 15 years, according Dutch legislation. No one besides the research team had access to the data.

## 3 FINDINGS

Fourteen ambulance nurses participated in this study. Nine participants were male. The average age of the participants was 50 years (range, 38–61), and the average years of working experience as an ambulance nurse was 16 years (range, 5–27). See Table 2, Demographic characteristics. Eleven interviews took place at the ambulance station, and three interviews took place at the home of the participants. Theoretical saturation was reached after 12 interviews. Two extra interviews took place to confirm saturation. The interviews lasted 47 min on average.

**Table 2:** Demographic characteristics

Participant	Regional ambulance service location	Gender	Work experience as ambulance nurse (in years)
1.	A	Female	8
2.	A	Male	22
3.	A	Male	22
4.	B	Female	18
5.	A	Female	8
6.	A	Male	16
7.	C	Male	18
8.	C	Male	26
9.	D	Male	7
10.	E	Female	18
11.	E	Male	15
12.	E	Male	5
13.	C	Female	10
14.	B	Male	27

### 3.1 Two main themes emerged from the data

The first theme is “It is not my cup of tea, but some like it.” It characterized the beliefs of participants on persons with psychiatric disorder, mental health care, and their professional identity relating to the emergency care for patients with manic and/or psychotic symptoms.

The second theme is “You never know what you're gonna get.” This theme describes tasks and responsibilities of ambulance nurses, multidisciplinary collaboration, and the experience of transporting patients with acute manic and/or psychotic symptoms. Some subthemes overlapped.

### 3.2 Theme 1: It is not my cup of tea, but some like it

#### 3.2.1 It is a different world

A minority of participants described their experiences with mental health as “interesting” and “fascinating.” Most referred to it as elusive and a “different world.”

*“*Psychiatry felt unsatisfactory, because you can't help the patient. You don't see that the patient is getting better.*”*

Most participants expressed uncertainty about an ambulance being the most suitable means of transport for patients with acute manic and/or psychotic symptoms.

*“*The ambulance contains a lot of medical equipment. It's a restless environment.*”*

Participants stated that they were insufficiently trained to guide persons with psychiatric disorder.

*“*In our education as a nurse, we learned something about psychiatry. But this was very limited; we just have too little knowledge.*”*

Many participants explained that in emergency care the focus is on quickly assessing the condition of the patient, immediately followed by adequate interventions. In their view, in (emergency) psychiatric care the focus is on communication and taking time; this does not fit with their regular way of working.

*“*As an ambulance nurse, I am used to action and quick interventions. But with these patients, sometimes you have to use the handbrake…. Going too fast may have the opposite effect.*”*

#### 3.2.2 I am the captain of my ship

Participants characterized their work as practical, quick fixes, and solving problems. They saw themselves as a short link between the mobile crisis team and clinical care. When on call, participants collect as much information about the patients as possible, describing it as having a de‐escalating effect on the patient somehow. Participants expressed the need to follow their “gut feeling.”

*“*The mobile crisis team can say that it's safe to transport the patient, but that doesn't interest me. I will take it into account, but it isn't decisive for me. I make my own decision; it's my transport, not theirs.*”*

Participants mentioned that a relaxed attitude, clear communication, and taking time to act are important during their work with persons having psychiatric disorder. Most of the participants expressed trying to be themselves and communicating on the same level as the patient. Limiting stimuli was considered important during transport.

*“*I dim the light, no music, no distracting things. It's important to stay calm, both verbally and physically.*”*

There were contrasting perspectives on how competent participants felt in psychiatric emergency care. When participants mentioned feeling competent, they mostly said this was caused by having work or personal experience in mental health care.

*“*As a child, I lived on the grounds of a psychiatric hospital. Psychiatry is ‘everyday habits’ for me.*”*

When participants stated that they were feeling incompetent, they experienced a lack of education and sometimes feelings of fear for these patients.

*“*Our interventions after a car accident are clear; the approach is structured. Psychiatric disorders are harder to understand. You cannot learn them from paper.*”*

#### 3.2.3 It can be freaking scary

Some participants described feeling uncomfortable transporting a patient with acute manic and/or psychotic symptoms. They were sitting alone with the patient in a literal small space in the ambulance.

*“*You can't go anywhere. You can't evade. You can't leave; nobody can leave. That makes it risky; that's hard.”

Participants mentioned that they have a feeling that other involved professionals were not aware that ambulance nurses are alone with the patient during transport in the ambulance.

*“*When we arrived at the psychiatric hospital, four to six people were waiting. I was alone with the patient.*”*

### 3.3 Theme 2: You never know what you're gonna get

Ambulance nurses described two scenarios about how they were involved in care for patients with acute manic and/or psychotic symptoms. Scenario A: The ambulance was the first on the scene. Ambulance nurses made executive decisions and proceeded to care. Scenario B: The mobile crisis team was already involved, calling an ambulance specifically for the transport of the patient to a psychiatric hospital.

#### 3.3.1 Going in and doing your thing

In the case of Scenario A, participants described trying to create an overall picture of the patient to indicate what care is needed.

*“*We collect as much information as possible. Did he use medication? Did he drink 20 bottles of beer? You try to get clear which route you should follow.*”*

In the case of Scenario B, nurses received the notification for transport from the control room. Participants described experiencing an unpleasant and unpredictable feeling when on route. This was partly determined by the limited information provided from the control room. When participants arrived at the location, they needed to be informed by professionals of the mobile crisis team. Some participants mentioned that it is hard to get information.

*“*Information can make or break our actions. Psychiatrists don't realize that. It feels like being just a taxi. That's a bit like a degradation of our profession.*”*

#### 3.3.2 Is there anybody out there?

In the case of Scenario A, there is a difference in consulting general healthcare specialists versus mental healthcare specialists, especially psychiatrists. Participants noted that a couple of years ago, it was impossible to contact mobile crisis teams directly. Nowadays, it is possible, which is seen as an improvement. Due to the optimization of consultation, participants experienced more understanding about the way mobile crisis teams act. Some participants noticed difficulties in collaboration. They stated sometimes feeling deserted by mobile crisis teams.

*“*Professionals of the mobile crisis team often said, ‘The deal was he shouldn't drink’. Now he drank, so we do nothing.*”*

When the mobile crisis team is unwilling or unable to respond and there is an acute need for care, patients were transported to the emergency department (ED). However, participants expressed that persons with psychiatric disorder do not belong in an ED.

*“*Psychiatric patients are sitting in a booth, start walking back and forth, and ask for attention, but that kind of attention is hard to give in an ED.*”*

Another option the participants used is consulting the general practitioner (GP) by telephone to guarantee care. Thereby, they try to create support for the patient. Generally, participants have little contact with family members of persons with psychiatric disorder, partly due to the small social network many persons with psychiatric disorder have. When family members are involved, participants try to estimate what influence they have on the patient's well‐being. Participants also expressed feeling frustrated when the mobile crisis team or a GP indicated that it was acceptable for ambulance nurses to leave the patient.

*“*You are afraid of letting someone go who is possibly a danger to himself or his environment.*”*

In the case of Scenario B, participants stated that the course of action was clearer and experienced the collaboration as more effective. However, participants mentioned that even in this case, effort has to be made to get sufficient information.

*“*The legal correspondence is faxed to the control room; we don't get that information. That's a shame.*”*

Participants mentioned that when they want to evaluate their work, they could go to a so‐called “relief team,” which is unusual to do. The purpose of the relief team is to evaluate the provided care in a specific case, to share experiences and thus to conclude the case.

Meanwhile, conversing with colleagues during coffee breaks was more common and was experienced as helpful. Other than “coffee‐break consultation,” multidisciplinary debriefings rarely happen, according to participants. A lack of money, staff shortage, and differences within organizations make it complex.

#### 3.3.3 Mind your step

In both scenarios, the assessment of safety was mentioned as an important task before transporting a patient. Participants stated that the assessment consisted of collecting information from family members, police, and/or the mobile crisis team. Thereby, the behavior and the cooperativeness of the patient were seen as important indicators for decision making. When participants felt they could not guarantee their own safety or that of the patient, the mobile crisis team was asked to administer a sedative before transport. However, participants noted that the mobile crisis team is reluctant on this topic.

*“*I asked the mobile crisis team, ‘Did he take his medication?’ ‘No, he didn't. He won't take it’. The mobile crisis team respects the autonomy of the patient, while we think the transport has to be safe.*”*

Another option is to call for assistance from police or security guards and/or using restraints during transport. When police are involved to reduce the risk of dangers, or disruptive behaviors in Scenario A, some participants experienced that the police had another approach, stricter and more heavy‐handed, which, in some cases, evoked more aggression.

*“*The police are always really quick to be heavy‐handed, force a patient down on the floor, and use handcuffs. I think this can be done in a different way.”

When police or security guards provide assistance in Scenario B, they could drive behind the ambulance or sit in the ambulance. This depends on the wishes of ambulance nurses. Involvement of the police gave participants a feeling of safety.

#### 3.3.4 Transport exceptionnel

Some participants experienced that transport of patients with acute manic and/or psychotic symptoms could feel uncomfortable.

*“*When someone is staring at you for 45 minutes and says nothing, that doesn't feel good at all.*”*

Collaboration with ambulance drivers was seen as important, although the interpretation of this collaboration varied. Some participants mentioned they debated with drivers about a plan of action before transport. Others mentioned the importance of having a driver in the background, silent but alert.

*“*I communicate with the driver through the mirror. One facial expression says it all.*”*

Participants described having different preferences in the means of transport. Some participants mentioned always transporting patients on a stretcher. Other participants mentioned preferring persons with psychiatric disorder sitting in a chair, because they believe this way of transport is less threatening for these patients.

## 4 DISCUSSION

This study explored the experiences of Dutch ambulance nurses in emergency care for patients with acute manic and/or psychotic symptoms. The theme “It's not my cup of tea, but some like it” referred to the beliefs of participants on persons with psychiatric disorder, mental health care, and their professional identity relating to emergency care for patients with manic and/or psychotic symptoms. Participants (a minority) who saw mental health as “interesting” frequently felt competent. In contrast, many participants who saw mental health care as a “different world” felt incompetent and uncomfortable due to a lack of education and/or feelings of fear in caring for persons with psychiatric disorder. Participants indicated that the transport of patients with acute manic and/or psychotic symptoms rarely occurs. Thus, feelings of unfamiliarity could be experienced. A recent Australian study about the experiences of ambulance nurses in care for men with mental health or substance abuse problems suggested that feelings of incompetence were caused by the focus of ambulance nurses’ education on the physical aspect.^10^ It is possible that the patient notices the ambulance nurse feelings of uncertainty and incompetency in dealing with acute symptoms of mania or psychosis evoking or increasing feelings of unsafety or anxiety.

The theme “You never know what you're gonna get” is characterized by the description of tasks and responsibilities of ambulance nurses, multidisciplinary collaboration, and the experience of transporting patients with acute manic and/or psychotic symptoms. When the ambulance was the first on the scene, ambulance nurses made executive decisions and proceeded to care. In that case, a lot is being asked of ambulance nurses. They must be able to identify psychiatric symptoms, while participants mentioned that they are not adequately trained to do that.

In the basic nursing training in The Netherlands, psychiatry is rather a limited part of the curriculum. Most of our participants, as can be seen in Table 2, followed this training a long time ago. Persons with psychiatric disorder were often referred to an ED. An Australian study examined the effects of a specialized mental health team (SMHT) on the length of stay of persons with psychiatric disorder in an ED.^18^ The presence of a SMHT increases pressure and minimizes the length of stay of persons with psychiatric disorder in an ED. Participants in our study mentioned that it can be hard to arrange appropriate psychiatric care when they are the first on the scene. This finding is in line with experiences of ambulance nurses in Australia: Faddy et al.^19^ investigated the effects of a mental health acute assessment team (MHAAT). This team consisted of a specialized mental health nurse and a paramedic. The MHAAT assessed the need of care at home, offered follow‐up care within 3 days, or referred and transported the patient to a psychiatric hospital if needed. The study rated MHAAT as highly successful; 69% of the patients were able to refer to a destination other than EDs or did not need transport.^19^ Although our study focused only on emergency care for patients with manic and/or psychotic symptoms, the findings are in line with studies that focused on the experiences of ambulance nurses with general mental health care.^10^, ^11^, ^12^

### 4.1 Strengths and limitations

This study has several strengths. First, five RAVs in the eastern Netherlands were included to compile a varied range of experiences, which enhances the transferability.^15^ Second, more than half (*n* = 8) of the participants reacted on the member check, as described in Section 2.4. Using a member check enhances the credibility.^15^, ^16^ Third, theoretical saturation was reached, and with the use of peer debriefing, bias or inappropriate subjectivity was detected.^15^

This study also has some limitations. First, most participants have many years of work experience as ambulance nurses (on average, 16 years). This makes the sample unrepresentative for ambulance nurses and may affect transferability. Second, ambulance nurses were mainly recruited by managers. Ambulance nurses with less affinity for persons with psychiatric disorder might not have been asked or willing to participate in this study, which could lead to selection bias and is a limitation in generalizability. Third, we included only ambulance nurses in the eastern Netherlands, which influenced the generalizability. Participants mentioned that psychiatric emergency care in other parts of The Netherlands is better organized. Therefore, to create a complete view of experiences of Dutch ambulance nurses, it is recommended to examine experiences in all parts of The Netherlands.

## 5 CONCLUSIONS

Ambulance nurses in emergency care of patients with acute manic and/or psychotic symptoms often experience stress and uncomfortable feelings due to different factors: a lack of information on the patients, being alone with the patient in a small space and the unpredictability of the situation. Besides the collaboration and communication with the other professionals in the chain of emergency care is not always optimal.

## 6 IMPLICATIONS FOR NURSING PRACTICE

Reduction of stress and uncomfortable feelings could be achieved when ambulance nurses are better informed about patients by other involved professionals. The use of transfer documents could be helpful: in this document the acute psychiatric condition of the patient is explained. Feelings of incompetence about mental health care can be addressed by education about disorders and used interventions, as well as practice simulation, in a continuous learning process. It is important to learn together with other professionals involved in interdisciplinary consultation and make agreements about collaboration and the performance of care. In this way, the quality of psychiatric emergency care could be improved.

## CONFLICT OF INTERESTS

The authors declare that there are no conflict of interests.
